# A Comparative Study of Phenolics in Green Husks of Selected Hungarian Walnut Cultivars

**DOI:** 10.3390/plants15081245

**Published:** 2026-04-17

**Authors:** Laurine Kithi, Enikő Horváthné Szanics, Mária Berki, Éva Lengyel-Kónya, Rita Tömösközi-Farkas, Eszter Benes, Gitta Ficzek, Verina Krasniqi, Geza Bujdosó

**Affiliations:** 1 Research Centre for Fruit Growing, Hungarian University of Agriculture and Life Sciences, 1223 Budapest, Hungary; kithi.laurine.betty.riziki@phd.uni-mate.hu; 2 Department of Food Chemistry and Analytics, Hungarian University of Agriculture and Life Sciences, 1223 Budapest, Hungary; horvathne.szanics.eniko@uni-mate.hu (E.H.S.); berki.maria@uni-mate.hu (M.B.); lengyelne.konya.eva@uni-mate.hu (É.L.-K.); tomoskozine.farkas.rita.adel@uni-mate.hu (R.T.-F.); benes.eszter.luca@uni-mate.hu (E.B.); 3 Department of Fruit Growing, Institute of Horticultural Sciences, Hungarian University of Agriculture and Life Sciences, Villanyi u. 29-43, 1118 Budapest, Hungary; ficzek.gitta@uni-mate.hu (G.F.); krasniqiverina@gmail.com (V.K.)

**Keywords:** *Juglans regia* L., green husks, phenolic compounds, variations

## Abstract

Green husks, which are the fleshy pericarp of *Juglans regia* L. fruit, are an abundant yet under-utilized source of bioactive compounds. They are useful for plant defense and have potential for valorization to multiple commercial products. This study characterized total phenolic content (TPC) and individual phenolics in green husks of four Hungarian-bred cultivars (Milotai 10, Milotai intenzív, Milotai kései, Esterhazy kései) and one U.S. cultivar (Chandler). Phenolic compounds were extracted with aqueous organic solvents, quantified by HPLC-DAD and qualitatively identified by HPLC-MS. Linear mixed-effects models were used to assess the effects of cultivar, year, sampling time, and cumulative growing degree days (GDDs) on TPC and compound profiles. Mean TPC ranged from 34.9 to 57.2 mg GAE g^−1^ DW, with significantly higher values in the warmest year, 2024, and in cultivar Esterhazy kései compared with Chandler. Across cultivars and years, phenolic levels were generally elevated at early lignification (S1, BBCH 73–75) and at full maturity (S5–S6, BBCH 87–88), with depressed concentrations during mid-fruit development (S2–S4, BBCH 77–86). Several hydroxycinnamic acids, flavonoids, and naphthoquinones showed cultivar-specific and year-dependent patterns. Thermal conditions (cumulative GDDs) explained a substantial proportion of residual variation in TPC. These results highlight the combined roles of genotype, seasonal climate, and developmental stage dependencies in biosynthetic processes of phenolics in walnut green husks despite the diversity in factor effects.

## 1. Introduction

Over the last two decades, the global area under walnut cultivation has increased from 608,147 ha in 2001 to 1,247,938 ha in 2022, accompanied by a rise in in-shell production from 1.33 to 3.87 million tons [1,2]. With increasing production, by-products such as green husks and shells are generated in large quantities. Botanically, the green husks constitute to the fleshy pericarp (exocarp and mesocarp) covering the shell and kernel of the drupe-like walnut fruits. Dry green husks account for roughly 17–34% of the dry inshell biomass, representing a substantial lignocellulosic and phytochemical resource [3].

Walnut green husks are rich in bioactive phenolic compounds like phenolic acids, flavonoids, and naphthoquinones [4,5,6,7,8,9,10]. These compounds are synthesized through the shikimate and phenylpropanoid pathways under the control of specific enzymes and transcription factors modulated by abiotic and biotic stresses [11,12,13]. Comparative analyses of bark, leaves, and green husks indicate that husks generally contain the highest phenolic levels [14]. Moreover, both phenolic profiles and overall total phenolic content (TPC) vary with cultivar, origin, and phenological stage, as well as extraction solvent, farming practices, and agro-ecological conditions [15,16].

Plant polyphenols contribute to antimicrobial, antifungal, allelopathic, and antioxidant defenses, thereby supporting plant adaptability and resilience [17,18]. In walnut, phenolics in green husks have been linked to enhanced tolerance to *Xanthomonas arboricola* pv. *juglandis* [19,20]. Juglone (5-hydroxy-1,4-naphthoquinone), abundant in husks, has the potential to suppress the virulence of the root-knot nematode Meloidogyne hispanica and exhibits allelopathic effects on several plant species, a potential natural herbicide [21,22,23]. Juglone-rich extracts have also been used as eco-friendly dyes for natural and synthetic fibers because of their characteristic brown pigmentation [24].

Phenolic compounds from walnuts are additionally relevant for human health. Their antioxidant and anti-inflammatory properties contribute to protection against oxidative damage and have been associated with reduced risk or progression of metabolic, cardiovascular, and neurodegenerative disorders, as well as certain cancers [15,25,26,27,28,29,30]. Flavonols such as quercetin, myricetin, and kaempferol have been investigated as chemopreventive agents for various cancers and in type 2 diabetes [31,32]. Juglone from green husk extracts exerts antiproliferative effects on HL-60 leukemia cells, with apoptosis observed at 10 μm, highlighting its potential as a lead compound for anticancer therapy [33]. Phenolic acids, including gallic, ellagic, chlorogenic, syringic, and caffeic acid, as well as catechin and epicatechin, have also shown promising effects in models of inflammatory bowel disease, cardiovascular dysfunction, hypertension, and neurodegeneration [34,35].

Despite the abundance of bioactive compounds in green husks, they remain an underutilized by-product in most production systems. Green husks represent an important organic material for multiple industrial applications, thus a critical organ to examine and understand how phenolic biosynthesis responds to genotype and environment. Most existing studies have focused on single cultivars, one-season sampling, or post-harvest material, providing limited insight into how cultivar, seasonal climate, and fruit developmental stage jointly affect phenolic profiles under orchard conditions. Thus, the temporal dynamics of individual phenolic classes in relation to thermal conditions and key phases such as early lignification and nut maturity remain poorly described. In this study, we examine total phenolic content and major phenolic compounds in green husks of four Hungarian Persian walnut cultivars (Milotai 10, Milotai intenzív, Milotai kései, Esterhazy kései) and the widely grown cultivar ‘Chandler’ over three production seasons, and evaluate the effects of cultivar, year, and sampling time on phenolic accumulation, including the effect of thermal energy (cumulative growing degree days). Characteristics of the cultivar under investigation are summarized in Table 1 below.

## 2. Results

### 2.1. Cumulative Growing Degree Days

Based on the temperature data obtained, the cumulative thermal energy recorded at each sampling time was evaluated and presented in Table 2. The evaluation revealed higher thermal energy during sampling time in 2024, followed closely by 2022. The year 2023 was the coolest of them all. In the warmer years (2024 and 2022), cultivars reached maturity at different times.

### 2.2. Total Phenolic Content

Descriptive statistics of total phenolic content in each cultivar during the three production seasons (2022–2024) are indicated in Table 3. TPC ranged from 34.9 to 47.4 mg GAE/g DW in 2022. In 2023 and 2024, it ranged from 39.9 to 46.4 mg GAE/g DW and 50.0 to 57.2 mg GAE/g DW, respectively.

The study revealed (*p* < 0.05) a significantly higher TPC in 2024 (Y3), with an estimated average of 53.4 mg GAE/g DW, than in 2022 (42.6 mg GAE/g DW) and 2023 (42.1 mg GAE/g DW). Esterherzy kései had the highest TPC in 2024 (57.2 mg GAE/g DW) and 2022 (47.4 mg GAE/g DW). However, in 2023, Milotai 10 had higher TPC (46.4 mg GAE/g DW) compared to the other cultivars.

**Figure 1:**
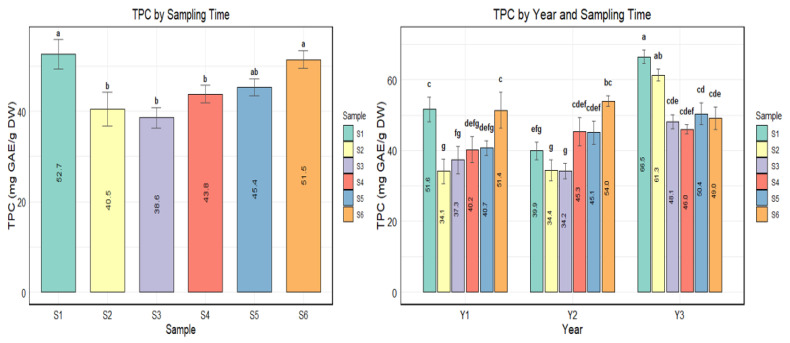
Total phenolic content (TPC, mg GAE/g DW) in green husks at different sampling times (S1–S6) in left panel and years (Y1–Y3) in right panel. Bars represent means ± standard error (*n* = 86). Data were analyzed using one-way ANOVA for sampling time (left panel) and two-way ANOVA with interaction (right panel). Significantly different samples (*p* < 0.05) are identified using Duncan’s multiple range test at α < 0.05. Tukey’s HSD post hoc test; different letters above bars indicate statistically significant differences among samples within a panel, while bars sharing at least one letter are not significantly different. Regression analysis, Table 4, indicated a significant cultivar effect on total phenolic content, TPC. Esterhazy kései showed significantly higher TPC (estimate = 4.74, *p* < 0.05) than the reference cultivar, Chandler. Other cultivars were not statistically different. Year effects were also modest, with TPC in 2023 similar to 2022, but a significantly higher TPC in 2024 (estimate = 9.31, *p* < 0.05) than in the first sampling year, 2022. Sampling time had a marginal effect on TPC. Relatively lower concentrations were evident in samples collected from mid-July, S2, to mid–late September, S6 (fruit maturity), compared to the early fruit development stage (late June, S1), particularly in late July, (S3) (estimate = −13.07, *p* = 0.051). As a random effect, thermal conditions (Cum_GDD) also influenced variability in total phenolic content substantially (variance 44.1 vs. residual 41.7).

Significant variations in TPC across sampling times were also evident. Samples collected during early lignification and fruit maturity stage had elevated TPC relative to samples collected in the mid-fruit development stages, Figure 1. The left panel of Figure 1 shows higher phenolic content in samples collected in late June (S1—lignification stage) and mid–late Sep (S6—full maturity), estimated at 52.7 mg GAE/g DW and 51.5 mg GAE/g DW, respectively.

**Table 4:** Linear mixed-effects regression model for total phenolic content (mg GAE/g DW).

Term	Coefficient	Std. Error	*t*-Value	*p*-Value
Intercept	48.146	4.986	9.657	0.000
E. Kesei	4.741	2.237	2.119	0.038
M.10	3.527	2.307	1.529	0.131
M. Intenzív	1.219	2.279	0.535	0.595
M. Kesei	−0.722	2.237	−0.323	0.748
2023 (Y2)	−1.027	4.084	−0.251	0.806
2024 (Y3)	9.310	4.007	2.323	0.039
Mid-July (S2)	−9.764	5.964	−1.637	0.131
Late July (S3)	−13.071	5.964	−2.192	0.051
Mid-Aug (S4)	−8.816	5.912	−1.491	0.166
Late Aug (S5)	−7.275	5.912	−1.231	0.246
Mid–Late Sep (S6)	−1.496	5.465	−0.274	0.789

Coefficients, standard errors, *t*-values and *p*-values from a linear mixed-effects regression function for TPC (mg GAE/g DW) with the response and cultivar, year, and sampling time as fixed effects. Cumulative growing degree days (Cum_GDD) is included as a random effect. Reference levels are Chandler (cultivar), 2022 (year), and late June (S1); *p*-value < 0.05 is considered statistically significant.

### 2.3. Phenolic Profiles in Persian Walnut Green Husks

The study identified 24 phenolic compounds in the four wavelengths; chromatographic images of these compounds are presented in Supplementary Materials Figure S1. They include hydroxybenzoic acids and derivatives (gallic acid, gallic acid derivative and gallic acid-hexoside, syringic acid derivative, and syringic acid-hexoside), hydroxycinnamic acids and derivatives (caffeoylquinic acid, neochlorogenic acid, caffeic acid-hexoside, coumaroylquinic acid, ferulic acid derivative, and ferulic acid derivative II), flavonoids (naringenin derivative, methoxynaringenin derivative, methylmyricetin derivative I, methylmyricetin derivative II, quercetin hexoside, methylmyricetin derivative, quercetin-pentoside, and quercetin-deoxyhexoside), naphthoquinones (juglone, dimethyljuglone, methoxy-juglone derivative, dihydroxy-juglone derivative, and dimethyl-bijuglone derivative). Table 5 presents the descriptive statistics for each compound and cultivar. It provides respective mean concentration ± standard deviation (µg standard equivalent g^−1^ DW) together with grouping letters from Duncan’s multiple range test (one-way ANOVA per compound, α = 0.05). Principal component analysis was done, and Figure 1 shows the PCA scores and phenolic loadings. The first three components in the original model explained 56.0% of the variance and in the validated models, they explained 47.7% of the variance, as shown in Table 6. Variation in phenolic profiles in PC1 (26.8%) was significant (*p* < 0.05) and was significantly influenced by cultivar, year of production, and sampling time. Variations in PC2 (16.6%) were largely driven by year effects, whereas variations in PC3 (12.8%) were driven by both cultivar and year effects.

**Figure 2:**
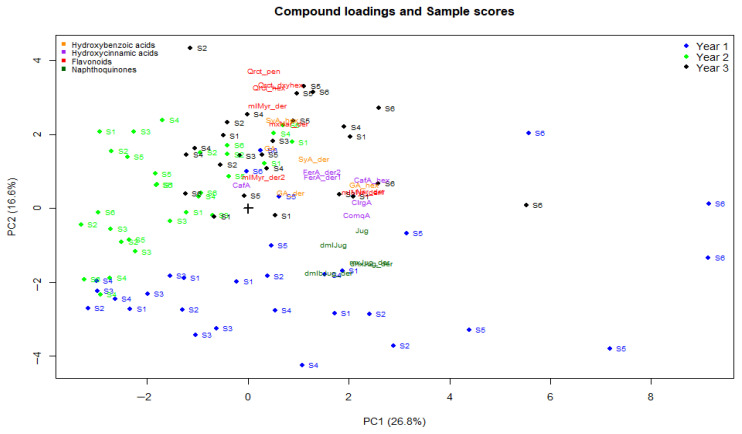
Biplot on phenolic loading and score clusters by year in PC1 and PC2. Note: Points represent individual samples colored by year; phenolic compounds are shown as loading labels scaled by a factor of six for graphical clarity. Therefore, interpretation focuses on the directions and relative positions of the compound.

The 2022 (Y1) scores clustered in the lower quadrants of the biplot (Figure 2) while the 2024 (Y3) scores clustered in the upper quadrants. The 2023 (Y2) scores overlap the two (top and lower) quadrants on the left. The 2022 sample cluster is evidently sparse compared to the 2023 and 2024 denser clusters. Flavonoids (quercetin glycosides, naringenin derivatives, and methylmyricetin derivatives) and hydroxybenzoic acids (gallic and syringic acid derivatives and glycosides), including some hydroxycinnamic acids (caffeic acid-hexoside and ferulic derivatives) dominated the upper right quadrant, suggesting dominance of these compounds in Year 3 (2024) samples.

**Figure 3:**
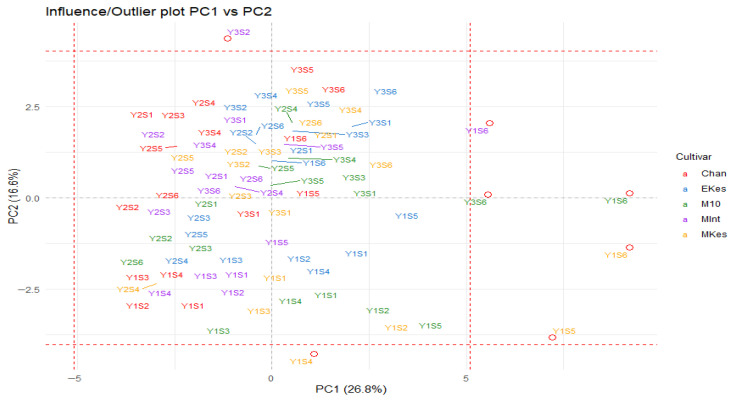
Outliers in PC1 and PC2.

In contrast, naphthoquinones (juglone and its derivatives) clustered in the lower right quadrant of the biplot, indicating elevated naphthoquinone concentrations compared to other compounds in the 2022 samples. Distinct cultivar clusters (Supplementary Materials, Figure S2) were also observed with phenolic loadings positioned away from Chandler, suggesting lower compound concentrations than in other cultivars. The outlier plot of PC1 vs. PC2, Figure 3, reveals that there were some extreme compound scores of the cultivars Milotai kései, Milotai 10, and Milotai Intenzív in August (S4 and S5) and September (S6), mostly in 2022 and a few in 2024.

**Figure 4:**
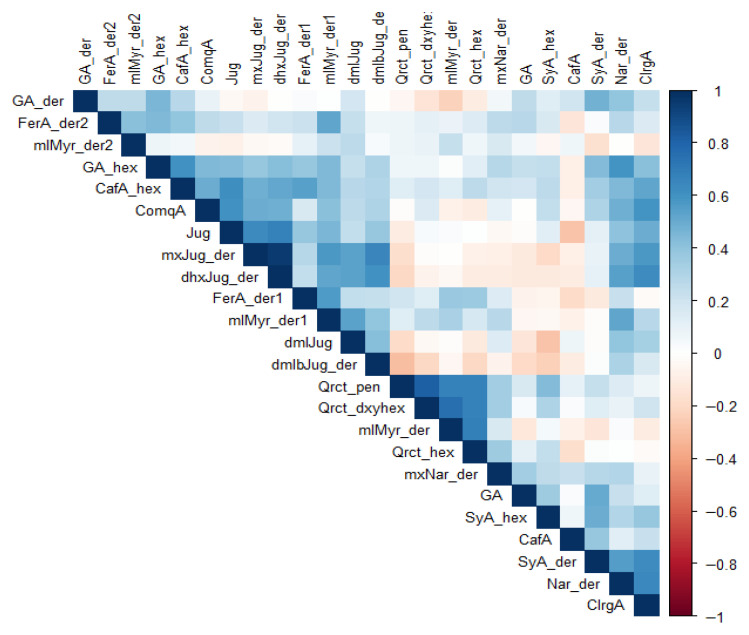
Correlation matrix of phenolic compounds. Short names and actual compound names: GA, gallic acid; GA_der, gallic acid derivative; GA_hex, gallic acid-hexoside; SyA_der, syringic acid derivative; SyA_hex, syringic acid hexoside; CafA, caffeic acid; CafA_hex, caffeic acid-hexoside; ClrgA, chlorogenic (caffeoylquinic) acid; ComqA, coumaroylquinic acid; FerA_der1, ferulic acid derivative I; FerA_der2, ferulic acid derivative II; Nar_der, naringenin derivative; mxNar_der, methoxynaringenin derivative; Qrct_hex, quercetin-hexoside; Qrct_pen, quercetin-pentoside; Qrct_dxyhex, quercetin-deoxyhexoside; mlMyr_der, methyl-myricetin derivative; mlMyr_der1, methyl-myricetin derivative I; mlMyr_der2, methyl-myricetin derivative II; Jug, juglone; dmlJug, dimethyl-juglone; mxJug_der, methoxy-juglone derivative; dhxJug_der, dihydroxy-juglone derivative; and dmlbJug_der, dimethyl-bijuglone derivative.

Similar to the biplot (Figure 2), in which biosynthetically related compounds are loaded in the same direction, the heatmap (Figure 4) revealed clusters of correlated compounds (compound codes are defined in Figure 4 caption). Some naphthoquinones (juglone and its derivatives), hydroxycinnamic acids (chlorogenic acid, syringic acid), and their esters (caffeoyl-, coumaroyl- and feruloyl-type), and the flavonoids (quercetin and methylmyricetin glycosides) were positively correlated.

**Figure 5:**
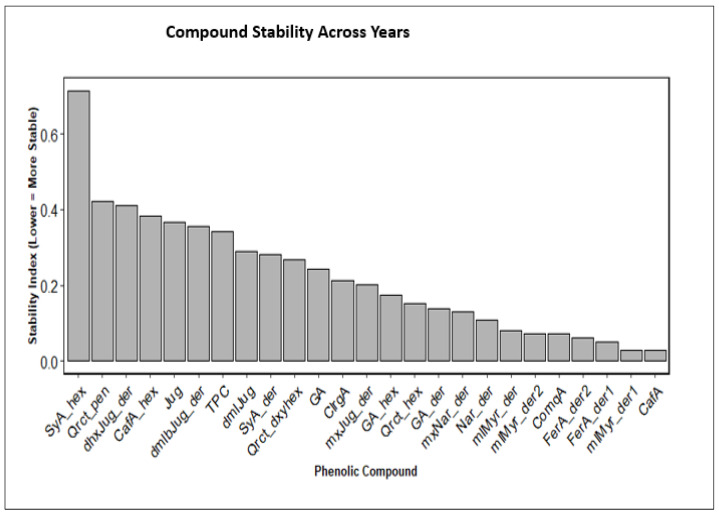
Compound stability across years.

Some naphthoquinone derivatives (juglone, dimethyljuglone, and dimethyl-bijuglone derivative) showed moderate negative correlations with some phenolic acids (gallic acid, syringic acid hexoside, and caffeoylquinic acid) and flavonoid glycosides (methoxynaringenin derivative and quercetin-pentoside), indicating that increased concentration of these naphthoquinones decreases biosynthesis of these phenolic acids and flavonoids. The antagonistic behavior aligns with their characteristic opposite orientations in the PCA biplot, supporting the idea that contrasting phenolic profiles (juglone-rich vs. phenolic-acid/flavonoid-rich) characterize different subsets of samples or growing conditions. Further evaluation revealed that some phenolics are more stable than others, as demonstrated in Figure 5. Caffeoylquinic acid, methylmyricetin derivatives, ferulic acid derivatives, and coumaroylquinic acid showed the lowest temporal variability across seasons, suggesting comparatively high stability compared to syringic acid, quercetin, juglone, and their associates.

### 2.4. Factor Effects on Phenolic Compounds

Using a generalized linear model (GLM), we examined the effect of cultivar, year and standardized cumulative growing degree days on biosynthesis of phenolic compounds. Statistical significance of factor effects was assessed using Wald tests. Table 7 presents the effects of cultivar, growing season (year), and scaled cumulative growing degree days on biosynthesis of naphthoquinones. Supplementary Materials Table S1 provides factors effects on phenolic acids and flavonoids identified in the study.

#### 2.4.1. Effect of Thermal Energy (Cumulative Growing Degree Days)

The results indicate compound-specific associations with thermal energy (GDD). An overall positive effect on naphthoquinones was observed with juglone and dimethyl-bijuglone derivative concentrations strongly influenced (*p* < 0.05), suggesting higher concentrations at higher thermal conditions. Significant positive thermal energy effects (*p* < 0.05) were also seen in gallic acid-hexoside, methoxynaringenin derivative, caffeic acid-hexoside, ferulic acid derivative II, methylmyricetin derivative I, methylmyricetin derivative II, quercetin hexoside, and quercetin-deoxyhexoside. In contrast, gallic acid derivative and caffeoylquinic acid had a significant decline (*p* < 0.05) with increasing heat accumulation.

#### 2.4.2. Cultivar Effects

Hungarian cultivars presented relatively higher concentrations of naphthoquinones than Chandler, except Esterhazy kései, whose juglone concentrations were comparatively lower. Some flavonoids and phenolic acid conjugates, like gallic acid derivative, gallic acid-hexoside, naringenin derivative, chlorogenic acid, and syringic acid derivatives, were significantly higher in Hungarian walnuts than in Chandler (Supplementary Materials, Table S1).

#### 2.4.3. Growing Season (Year) Effect

Concentrations of all naphthoquinones were significantly lower in 2023 and 2024 relative to 2022, indicating a strong negative year effect on these phenolic compounds. Among flavonoids, biosynthesis of naringenin derivatives was significantly higher in 2024 compared to 2022, even though these compounds were lower in 2023 relative to 2022. Quercetin derivatives (quercetin hexoside, quercetin pentoside, and quercetin-deoxyhexoside) showed consistently higher concentrations in 2023 and 2024 than in 2022, suggesting enhanced accumulation of these glycosides in later seasons. By contrast, methylmyricetin derivatives I and II declined significantly in 2024, pointing to divergent year responses among flavonoids. For phenolic acids, gallic acid derivatives were significantly reduced in 2023 but increased in 2024. Syringic acid derivatives exhibited significantly higher concentrations in both 2023 and 2024 than in 2022.

## 3. Discussion

Biosynthesis of phenolic compounds in walnut green husks was influenced by multiple factors. Temporal environmental conditions during the production season, phenological stage of the fruit, and genetic differences influenced phenolic compound synthesis among the five cultivars studied. Phenolic compounds significantly varied across cultivars, with Esterherzy kései (48.7 ± 9.8 mg GAE/g DW) and Milotai 10 (47.5 ± 9.4 mg GAE/g DW) having higher concentrations than Milotai Intenzív, Milotai kései, and Chandler. Wu et al. [42], Cosmulescu et al. [43], and Sarikhani et al. [44] associate the significant variations in total phenolic content with cultivar differences.

The results in this study are similar to those of Cosmulescu & Trandafir [14], who established a significant effect of sampling time on total phenolics. Accumulation of total phenolics was characterized by peaks in early lignification stages in late June and July and during the maturity period in September, with depressed levels at mid-fruit development in late July and August. The total phenolic content at different sampling times during fruit development ranged from 38.6 to 52.7 mg GAE/g DW. These concentrations are relatively similar to the figures reported by Bujdosó et al. [19] of 44.2–57.4 mg GAE/g DW in Hungarian cultivars sampled during nut lignification under temperate Central European conditions and by Barekat et al. [4] of 35.2–59.8 mg GAE/g DW in Iranian cultivars collected at full ripening under a warm, semi-arid climate. In contrast, Akbari et al. [45] established comparatively low levels of 19.61–36.10 mg GAE/g DW in Iranian genotypes, whereas Bourais et al. [5] and Rahmani et al. [46] reported relatively higher quantities of 306.36 ± 4.74 mg GAE/g DW and 99.98–122.26 mg GAE/g DW, respectively. These discrepancies are likely attributable to differences in growing-season temperature regimes, extraction procedures, and genotype, among other factors. In our study, the highest TPC values occurred in 2024, the warmest year of the three seasons considered, suggesting that growing-season temperature, together with cultivar and developmental stage, influences the biosynthesis of total phenolic content.

**Figure 6:**
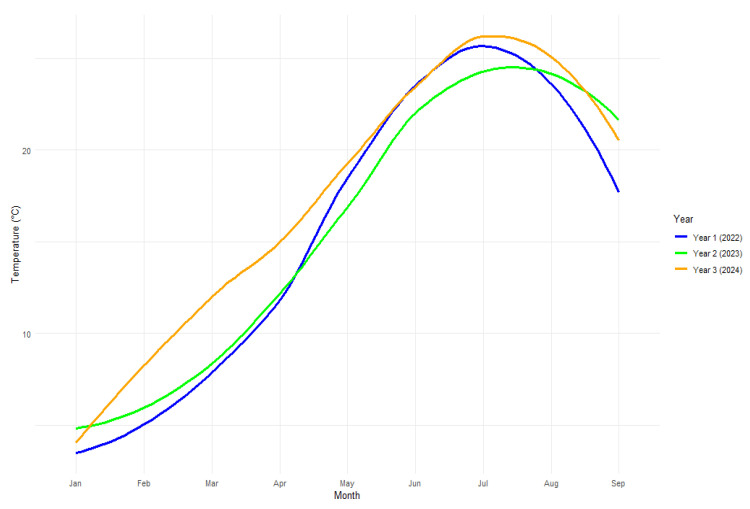
Average temperatures during sampling period, 2022–2024.

As observed in this study, significant shifts in total phenolics are attributed to the year of sampling. Total phenolics were higher on average by 55.3 mg GAE/g DW in 2024, which, as indicated in Figure 6, was evidently warmer than 2022 and 2023. These variations may be associated with the warmer growing season, among other temporal agroecological changes such as rainfall and agronomic practices. Wu et al. [47] reported a similar observation where variations in agroecological conditions influenced the accumulation of compounds in *J. regia* L.

The study identified 24 phenolic compounds comprising multiple flavonoids, hydroxycinnamic acids, hydroxybenzoic acids, and naphthoquinones occurring as simple phenolic acids, esters, glycosides, and other conjugated forms. The walnut husks contained gallic acid, gallic acid derivative, gallic acid-hexoside, syringic acid-hexoside, syringic acid derivative, caffeoylquinic acid, neochlorogenic acid, caffeic acid-hexoside, coumaroylquinic acid, two ferulic acid derivatives, naringenin derivative, methoxynaringenin derivative, three derivatives of methylmyricetin, quercetin hexoside, quercetin-pentoside, quercetin-deoxyhexoside, juglone, dimethyljuglone, methoxy-juglone derivative, dihydroxy-juglone derivative, and dimethyl-bijuglone derivatives occurring in different concentrations. Mates et al. [48], Bourais et al. [5], Medic et al. [7], and Pycia et al. [49] report the presence of similar compounds in diverse compositions.

Similar to TPC, accumulation of individual phenolic compounds was dependent on cultivar, year of sampling, and thermal energy. As demonstrated in the PCA biplot, distinct year-based sampling clustering suggests a strong year effect on phenolic accumulation. Related compounds clustered together as well. The biplot suggests highly variable but juglone-rich phenolic profiles in 2022. On the other hand, in 2024 (Y3), lesser variations were dominated by phenolic acids and flavonoids. The 2023 (Y2) ellipse was at an intermediate position, indicating that phenolic concentrations in this year were within average. However, the 2023 cluster vis-à-vis direction of phenolic loadings suggested inverse correlation with compound loading. This is consistent with the regression results, shown in Table 6, where the second sampling season (Y2) reported highly significant lower outcomes.

Congruent with PCA was the correlation matrix, with some naphthoquinones, hydroxycinnamic acids, and flavonoids exhibiting moderate positive correlation. Other clusters of phenolic acids and flavonoids were negatively correlated, consistent with biochemical trade-offs in biosynthetic pathways during fruit development [50]. Shifts in compound concentrations were characterized by peaks at early lignification and full maturity and depressed phenolic levels as the fruit enlarges in mid-development stages. A study of plums by Zang et al. [51] reveals similar results with phenolic compounds exhibiting higher concentrations at fruit maturity. Shifts in compound concentrations are associated with structural reinforcement, increased conversion of soluble phenolics to lignin, and a rigid polymer to reinforce cell walls for physical defense [51,52].

Caffeoylquinic, coumaroylquinic, ferulic acids, and derivatives, including methylmyricetin derivatives, exhibited minimal variability in concentrations across seasons. Xue et al. [53] published similar findings where some forms of chlorogenic acids remained stable except when subjected to high temperatures. In contrast, a higher stability index in juglones and syringic acid derivatives, the quercetins, may be attributed to higher oxidative activities associated with these compounds.

## 4. Materials and Methods

### 4.1. Trial Farm and Sample Collection

Walnut fruit samples were collected from the Experimental Fields of the Hungarian University of Agriculture and Life Sciences, Research Centre for Fruit Growing at Alvira major in Érd, Hungary. GPS coordinates of the sampling site are 47°20′11.44″ latitude and 18°51′53.42″ longitude. The orchard was established in 1990 with grafted seedlings planted at a spacing of 10 m by 10 m and trained to a central leader canopy system. It is managed under rainfed conditions, and has chernozem soil with high lime (pH = 8, total lime content in the top 60 cm layer 5%) and humus (2.3–2.5%) content. The soils have medium compactness (KA = 40) based on the Arany-type cohesion index.

The average annual temperature during the trial period (between 2022 and 2024) was 12.18 °C. During the growing season (between March and September), the average temperatures were 16.97 °C. During the spring months (March to May), the average minimum temperatures were 4.31 °C, with the number of frost days recorded as 7 days. The annual precipitation during the 3 years of sampling was between 276.6 and 323.3 mm/year (Table 8).

Average monthly temperature averages (Figure 6), including daily temperature from January to September of each sampling year (2022–2024), were also collected from a meteorological station near the sampling site in Budapest, Hungary

The daily temperature of each sampling date was used to compute the growing degree days (GDDs) as $GDD=\frac{T_{max}+T_{min}}{2}-T_{base}$, where *T_max_* and *T_min_* are the maximum and minimum temperatures, respectively, and *T_base_* is the base temperature. Based on Soleimani et al. [1] and Bujdoso et al. [54], we used a *T_base_* of 5.5 °C. The thermal calendar of each sampling date was computed as $cumGDD=\sum_{l=1}^{n}\left(T_{av}-T_{base}\right)$, where *cum-GDD* is the cumulative GDD, n is the number of days from 1st January and Tav is the daily average temperature.

Five cultivars, Eszterházy kései, Milotai kései, Milotai intenzív, Milotai 10 and Chandler (control cultivar), were studied. Biweekly sampling was done from specific trees from the onset of the lignification period in mid-June to fruit maturity in mid-to-late September. In this study, we defined six sampling stages: late June (S1), mid-July (S2), late July (S3), mid-August (S4), late August (S5), and mid-to-late September (S6). Based on the principal growth stages of walnut phenology (BBCH Scale), S1, which is the early shell hardening and husk lignification, corresponds to BBCH 73–75, while S2–S4, which are the mid-fruit development and early fruit ripening stages, correspond to BBCH 77–86. S5–S6 are the full nut maturity stages and initial husk dehiscence, corresponding to BBCH 87–88 [54,55]. Green husks (pericarp) at each sampling time were peeled, lyophilized and blended into powder for chemical analyses.

### 4.2. Measurement of Total Phenolic Compounds (TPCs)

The Folin–Ciocalteu spectrophotometric method described by Singleton et al. [56] was used to determine the total phenolic content (TPC). For the extraction, 0.05 g of dried green husk powder was mixed with 10 mL of 80% (*v*/*v*) aqueous methanol (80% methanol, 20% bidistilled water; VWR International, Radnor, PA, USA), shaken thoroughly, and refrigerated for 24 h. After incubation, the mixture was shaken for an additional 30 min and subsequently filtered using a 0.45 µm PVDF syringe (Millipore, Burlington, MA, USA).

For the colorimetric reaction, 100 µL of the plant extract was mixed with 3.7 mL of distilled water and 0.5 mL of Folin–Ciocalteu reagent from Avantor (Radnor, PA, USA). After 1 min, 2 mL of 20% sodium carbonate solution was added. The mixture was then diluted to a final volume of 10 mL and incubated in the dark at room temperature for 1 h. Absorbance was measured at 750 nm. Gallic acid (Merck, Darmstadt, Germany) was used as the calibration standard, and TPC values were expressed as gallic acid equivalents (mg GAE/g DW) based on the dry weight of the plant extract.

### 4.3. Assessment of Phenolic Profiles

The phenolic profiles of the walnut green husk samples were analyzed according to the procedure of Nagy-Gasztonyi et al. [57]. In brief, the compounds were quantified by HPLC-DAD and qualitatively analyzed using an in-line single-quadrupole MS detector (HPLC-MS). A total of 100 mg of dried green husk powder was extracted with 10 mL of extraction solvent consisting of water and methanol containing 2% acetic acid (30/70, *v*/*v*). The mixture was sonicated at room temperature for 20 min, followed by centrifugation at 5000 rpm for 20 min. The resulting supernatant was then filtered through a 0.45 µm PVDF syringe filter.

For the chromatographic analysis, a Waters Alliance e2695 separation module equipped with a 2998 photodiode array detector (PDA) and Empower software (version 3.8.0) was used (Waters, Milford, CT, USA). The HPLC system was coupled to a Model Acquity Mass (QDa) detector (Waters, Milford, CT, USA).

Separation was achieved on a Sphinx column (5 µm, 250 × 4.6 mm; Macherey–Nagel, Duren, Germany) under gradient elution conditions, employing (A) 0.1% formic acid in water and (B) 0.1% formic acid in acetonitrile (HPLC grade; Merck, Darmstadt Germany) as mobile phases. The gradient elution program was as follows: 0–9 min, 5–20% B; 9–16 min, 20–22% B; 16–25 min, 22–50% B; 25–28 min, 50% B; 28–40 min, 50–100% B; 40–43 min, 100% B; 43–45 min, 100–5% B; and 45–50 min, 5% B. The injection volume was 20 µL, and the flow rate was set at 0.7 mL/min.

Mass spectrometric analysis was carried out in both negative and positive ionization modes. Electrospray ionization (ESI) was used as the ion source, and mass spectra were recorded in the m/z range of 100–1000. The probe temperature was maintained at 600 °C. In positive ion mode, the cone voltage was set to 15 V and the capillary voltage to 1.5 kV, whereas in negative ion mode the corresponding values were 50 V and 0.8 kV.

Identification of phenolic compounds was based on their spectral characteristics, retention times, measured mass-to-charge ratios (*m*/*z*), and fragmentation patterns. Identification of phenolic compounds was based on their spectral characteristics, retention times, measured mass-to-charge ratios (*m*/*z*), and fragmentation patterns. Quantification of the identified phenolic compounds was performed using calibration curves prepared from gallic acid at 280 nm, chlorogenic acid at 320 nm, quercetin at 355 nm, and juglone at 420 nm, all from Merck, Darmstadt, Germany.

### 4.4. Statistical Methods

The data were analyzed using RStudio version 2025.9.2.418 programming software. Shapiro–Wilk’s test and Levene’s test were used to check the normality of distribution and the homogeneity of variances, respectively. Factor effects on TPC were evaluated using a mixed-effects regression function, with cultivar, sampling time, and production season (year) as fixed effects and scaled thermal energy (Cum_GDD) as a random intercept. Post hoc comparison was done using Duncan’s multiple range test. Principal component analysis (PCA) was used to assess phenolic profiles. We also fitted linear models with log-transformed phenolic concentrations against cultivar, scaled-growing degree days, and production season (year) as fixed effects (log (concentration + 1) = cultivar + year + scaled cumulative GDD) to evaluate their role in biosynthesis of these compounds.

## 5. Conclusions

This study aimed to establish how cultivar, production season, fruit ontogeny, and thermal conditions influence total phenolic content and phenolic profiles in walnut green husks under orchard conditions. Across three sampling seasons (2022–2024), the TPC in green husks ranged from 35 to 57 mg GAE/g DW, confirming their importance for plant defense and as an agronomic by-product. The Hungarian cultivar ‘Esterhazy kései’ showed higher average TPC than ‘Chandler’, while the warmest year (2024) and the early lignification and nut-maturity stages were associated with elevated phenolic levels.

The study reveals that both the choice of cultivar and the timing of husk removal can be used as practical levers to influence the phenolic status of trees and the quality of husk by-products. Growing cultivars with generally higher TPC, such as ‘Esterhazy kései’, and targeting husk collection around early lignification and nut maturity could increase the availability of phenolic-rich material at dehulling. Because phenolic levels and profiles also shifted with year and cumulative growing degree days, local temperature patterns should be considered when planning harvest and making cultivar recommendations, especially under a warming climate. The cultivar- and year-specific composition of compounds also suggests that phenolic traits can inform breeding and management choices aimed at maintaining resilient and resource-efficient walnut production systems.

## Figures and Tables

**Table 1:** Origin and characteristics of walnut cultivars.

Cultivar	Origin	Harvest Time	Nut Diameter (mm)	Dried Nut Wt. (g/nut)	Kernel Color	Kernel Yield (%)
Milotai 10	Hungarian population	2nd–3rd week September	33–35	13–17	straw yellow	47–52
Milotai intenzív	Milotai 10 and Pedro hybrid	3rd week September	32–34	11–12	light yellow	55–57
Chandler	Pedro and UC 56-224 hybrid	3rd week September	28–30	12–13	light yellowish brown	49
Milotai kései	Milotai 10 and Pedro hybrid	2nd week October	32–34	12–15	light brown	42–45
Esterhazy kései	Hungarian population	3rd week of September	32–34	11–14	light brown	40–46

References: [6,36,37,38,39,40,41].

**Table 2:** Cumulative GDDs (°C days) at each sampling time during the three sampling seasons.

Year	Late June (S1)	Mid-July (S2)	Late July (S3)	Mid-Aug (S4)	Late Aug (S5)	Mid–Late Sep (S6)
2022	1439.6 ± 0.0	1515.2 ± 0.0	1788.2 ± 0.0	2124.1 ± 0.0	2358.9 ± 0.0	2749.0 ± 43.5
2023	1180.7 ± 0.0	1479.7 ± 0.0	1740.6 ± 0.0	1963.3 ± 0.0	2256.5 ± 0.0	2749.2 ± 0.0
2024	1450.3 ± 0.0	1750.0 ± 0.0	2082.1 ± 0.0	2359.7 ± 0.0	2666.9 ± 0.0	3151.1 ± 29.4

Note: Cumulative GDDs (°C days) evaluated from daily average temperature, a single observation per sampling date. Except for 2023, walnuts attained maturity at different dates with different daily average temperature in 2022 and 2024 giving non-zero SD.

**Table 3:** TPC (mean ± SD, mg GAE/g DW) by cultivar.

Year	Chan	E. Kes	M. 10	M. Int	M. Kes
2022	40.8 ± 11.8	47.4 ± 7.2	46.4 ± 12.7	43.2 ± 10.8	34.9 ± 3.2
2023	39.9 ± 8.6	41.7 ± 8.0	46.4 ± 7.8	41.9 ± 11.5	40.7 ± 11.0
2024	51.3 ± 10.2	57.2 ± 7.8	50.0 ± 7.9	53.0 ± 9.9	54.1 ± 11.4
Average	43.1 ± 10.7	48.7 ± 9.8	47.5 ± 9.4	45.6 ± 11.3	43.3 ± 12.1

**Table 5:** Descriptive statistics of phenolic compounds.

Compounds	Standard Equivalent (µg/g DW)	Chandler	E. Kesei	M10	M. Int	M. Kesei
Caffeoylquinic acid	Chlorogenic acid	7.7 ± 11.9 a	16.0 ± 16.2 a	13.6 ± 12.0 a	11.2 ± 11.4 a	13.2 ± 18.9 a
Caffeic acid-hexoside		47.2 ± 32.4 a	59.8 ± 24.5 a	63.4 ± 33.2 a	54.0 ± 29.8 a	58.2 ± 27.9 a
Neochlorogenic acid		420.5 ± 192.6 c	695.4 ± 343.9 b	1441.1 ± 629.7 a	732.8 ± 310.2 b	893.2 ± 365.5 b
Coumaroylquinic acid		513.4 ± 374.3 b	694.2 ± 313.0 b	1233.5 ± 624.7 a	767.5 ± 461.5 b	1326.1 ± 616.6 a
Ferulic acid derivative I		62.5 ± 56.1 ab	57.0 ± 19.9 ab	42.1 ± 15.8 b	51.8 ± 24.9 ab	67.1 ± 29.1 a
Ferulic acid derivative II		506.1 ± 523.4 b	2046.2 ± 1819.4 a	1052.5 ± 1023.1 b	853.5 ± 1303.2 b	868.3 ± 1143.8 b
Gallic acid	Gallic acid	171.3 ± 111.2 b	278.9 ± 238.3 a	102.2 ± 55.7 b	137.0 ± 104.6 b	99.2 ± 35.4 b
Gallic acid derivative		26.2 ± 32.1 b	124.3 ± 76.4 a	54.4 ± 36.2 b	38.7 ± 31.6 b	40.7 ± 39.7 b
Galic acid-hexoside		48.3 ± 28.9 b	87.2 ± 36.7 a	66.9 ± 37.0 ab	46.6 ± 31.9 b	78.4 ± 48.5 a
Naringenin derivative		211.2 ± 108.3 b	454.1 ± 157.1 a	455.0 ± 261.1 a	257.7 ± 92.8 b	465.2 ± 213.6 a
Syringic acid derivative		40.6 ± 36.9 b	136.6 ± 118.2 a	120.7 ± 59.4 a	90.5 ± 59.2 ab	118.0 ± 69.3 a
Syringic acid hexoside		246.4 ± 176.4 b	337.0 ± 246.7 ab	483.9 ± 348.0 a	341.9 ± 225.8 ab	321.1 ± 233.5 ab
Methoxy-naringenin derivative		385.6 ± 190.3 ab	441.6 ± 188.5 a	221.9 ± 148.5 c	306.0 ± 208.2 bc	444.4 ± 192.1 a
Quercetin-deoxyhexoside	Quercetin	158.1 ± 87.0 a	130.3 ± 62.0 a	156.9 ± 80.7 a	163.0 ± 88.5 a	150.9 ± 87.7 a
Quercetin-hexoside		644.2 ± 410.0 a	467.1 ± 234.3 ab	361.6 ± 261.3 b	573.4 ± 297.1 ab	468.5 ± 305.8 ab
Quercetin-pentoside		258.8 ± 148.7 a	251.4 ± 118.9 a	220.7 ± 128.3 a	259.4 ± 144.5 a	269.3 ± 141.1 a
Methyl-myricetin derivative		72.0 ± 48.1 a	44.2 ± 30.8 b	44.1 ± 31.4 b	61.9 ± 32.1 ab	56.1 ± 37.0 ab
Methyl-myricetin derivative I		19.8 ± 15.4 b	35.7 ± 26.7 ab	29.0 ± 26.9 ab	31.8 ± 25.1 ab	44.6 ± 38.0 a
Methyl-myricetin derivative II		42.1 ± 54.8 a	40.2 ± 61.6 a	10.6 ± 11.2 b	14.0 ± 23.7 ab	16.6 ± 17.1 ab
Dihydroxy-juglone derivative	Juglone	61.7 ± 44.6 c	82.5 ± 74.7 bc	391.1 ± 443.8 a	92.9 ± 89.4 bc	263.0 ± 350.8 ab
Dimethyl-juglone		66.4 ± 71.0 a	108.6 ± 106.8 a	111.2 ± 102.3 a	74.7 ± 64.7 a	118.1 ± 96.4 a
Dimethyl -bijuglone derivative		25.4 ± 22.8 b	59.8 ± 79.2 b	65.3 ± 62.9 b	39.7 ± 30.5 b	138.0 ± 180.2 a
Juglone		1124.8 ± 968.9 abc	674.0 ± 576.9 c	1815.6 ± 1345.4 a	982.4 ± 938.6 bc	1522.2 ± 1399.9 ab
Methoxy-juglone derivative		44.4 ± 39.7 b	55.0 ± 49.1 b	265.0 ± 351.4 a	71.2 ± 71.6 b	240.8 ± 377.4 a

Note: Values indicate mean ± SD of phenolic concentrations for all samples per cultivar (Chandler *n* = 16; Esterhazy kései and Milotai kései *n* = 18; Milotai 10 and Milotai intenzív *n* = 17) during the 3 years of sampling. Different letters within a row represent a significant difference among cultivars at *p* < 0.05 based on Duncan’s multiple range test in one-way ANOVA per compound; values sharing a letter are not significantly different.

**Table 6:** Variations explained by principal components.

PC	Cum. Explained Variance—Original	Cum. Explained Variance—Validated	*p*_Cultivar	*p*_Year	*p*_Sample
PC1	26.78	22.06	0.003	0.000	0.009
PC2	43.36	36.39	0.241	0.000	0.376
PC3	55.97	47.7	0.001	0.001	0.108

**Table 7:** Factor effects on log-transformed concentrations of naphthoquinones.

Phenolic Compound	Term	Estimate	Std. Error	*p*-Value
Dimethyljuglone	Intercept	4.330	0.213	0.000
	Esterhazy kései	0.426	0.255	0.099
	Milotai 10	0.491	0.258	0.060
	Milotai intenzív	0.203	0.258	0.433
	Milotai kései	0.612	0.255	0.019
	2023 (Y2)	−0.581	0.192	0.003
	2024 (Y3)	−0.780	0.204	0.000
	Scaled (Cum_GDD)	0.081	0.084	0.341
Methoxy-juglone derivative	Intercept	4.676	0.211	0.000
	Esterhazy kései	0.235	0.253	0.355
	Milotai 10	1.225	0.256	0.000
	Milotai intenzív	0.388	0.256	0.134
	Milotai kései	1.086	0.253	0.000
	2023 (Y2)	−2.235	0.190	0.000
	2024 (Y3)	−1.702	0.203	0.000
	Scaled (Cum_GDD)	0.053	0.084	0.528
Dihydroxy-juglone derivative	Intercept	4.934	0.180	0.000
	Esterhazy kései	0.093	0.215	0.665
	Milotai 10	1.426	0.218	0.000
	Milotai intenzív	0.278	0.218	0.206
	Milotai kései	0.936	0.215	0.000
	2023 (Y2)	−2.280	0.162	0.000
	2024 (Y3)	−1.072	0.172	0.000
	Scaled (Cum_GDD)	0.131	0.071	0.069
Juglone	Intercept	7.240	0.152	0.000
	Esterhazy kései	−0.378	0.182	0.041
	Milotai 10	0.653	0.184	0.001
	Milotai intenzív	−0.089	0.184	0.632
	Milotai kései	0.492	0.182	0.008
	2023 (Y2)	−1.553	0.137	0.000
	2024 (Y3)	−0.522	0.146	0.001
	Scaled (Cum_GDD)	0.296	0.060	0.000
Dimethyl-bijuglone derivative	Intercept	3.850	0.214	0.000
	Esterhazy kései	0.668	0.256	0.011
	Milotai 10	0.809	0.259	0.003
	Milotai intenzív	0.498	0.259	0.058
	Milotai kései	1.237	0.256	0.000
	2023 (Y2)	−1.121	0.193	0.000
	2024 (Y3)	−1.804	0.205	0.000
	Scaled (Cum_GDD)	0.266	0.085	0.002

Notes: Estimates, standard errors and *p*-values from GLM with Gaussian error and identity link (GLM) fitted separately for each compound, with cultivar, year and standardized cumulative growing degree days (scaled Cum_GDD) as fixed effects; *p*-value < 0.05.

**Table 8:** Monthly rainfall in millimeters (mm) during the sampling seasons (Érd, Elvira major, 2022–2024).

	Jan	Feb	Mar	Apr	May	Jun	Jul	Aug	Sep	Oct	Nov	Dec	Total
2022	7.0	11.6	8.0	61.1	18.8	54.6	11.9	43.7	125.1	8.3	34.3	59.2	323.2
2023	100.2	16.8	37.5	20.1	54.4	52.7	19.1	83.8	16.6	50.8	57.8	113.6	284.2
2024	25.5	23.3	8.4	25.5	43.8	59.9	33.2	25.3	80.5	37.3	19.7	24.0	276.6

## Data Availability

The original contributions presented in this study are included in the article. Further inquiries can be directed to the corresponding author.

## Supplementary Materials

The following supporting information can be downloaded at https://www.mdpi.com/article/10.3390/plants15081245/s1.
