# Supplementary material for: A Comparative Study of Phenolics in Green Husks of Selected Hungarian Walnut Cultivars
Source: Plants (Basel). 2026 Apr 17;15(8):1245. doi: 10.3390/plants15081245 (PMC13119689; doi:10.3390/plants15081245)
Supplement: Supplementary file 1 [file plants-15-01245-s001.zip › plants-4248076-supplementary.pdf]

A Comparative Study of Phenolics in Green Husks of Selected Hungarian Walnut Cultivars

Laurine Kithi [1], Enikő Horváthné Szanics [2], Mária Berki [2], Éva Lengyel-Kónya [2], Rita Tömösközi-Farkas [2], Eszter Benes [2], Gitta Ficzek [3], Verina Krasniqi [3] and Geza Bujdosó [1*]

[1.] Research Centre for Fruit Growing, Hungarian University of Agriculture and Life Sciences, Budapest, 1223, Hungary
[2.] Department of Food Chemistry and Analytics, Hungarian University of Agriculture and Life Sciences, Budapest, 1223, Hungary
[3.] Department of Fruit Growing, Institute of Horticultural Sciences, Hungarian University of Agriculture and Life Sciences, Villanyi u. 29-43, 1118 Budapest, Hungary
* Correspondence: geza.bujdoso@uni-mate.hu

Content

1. Figure S1: Chromatograms of compounds Identified in the 4 wavelengths (a. 280, b. 320, c. 355 and d. 420nm)
2. Figure S2: Biplot on phenolic loadings and scores by Culivar
3. Table S1: Factor Effects on Phenolic acids and Flavonoids

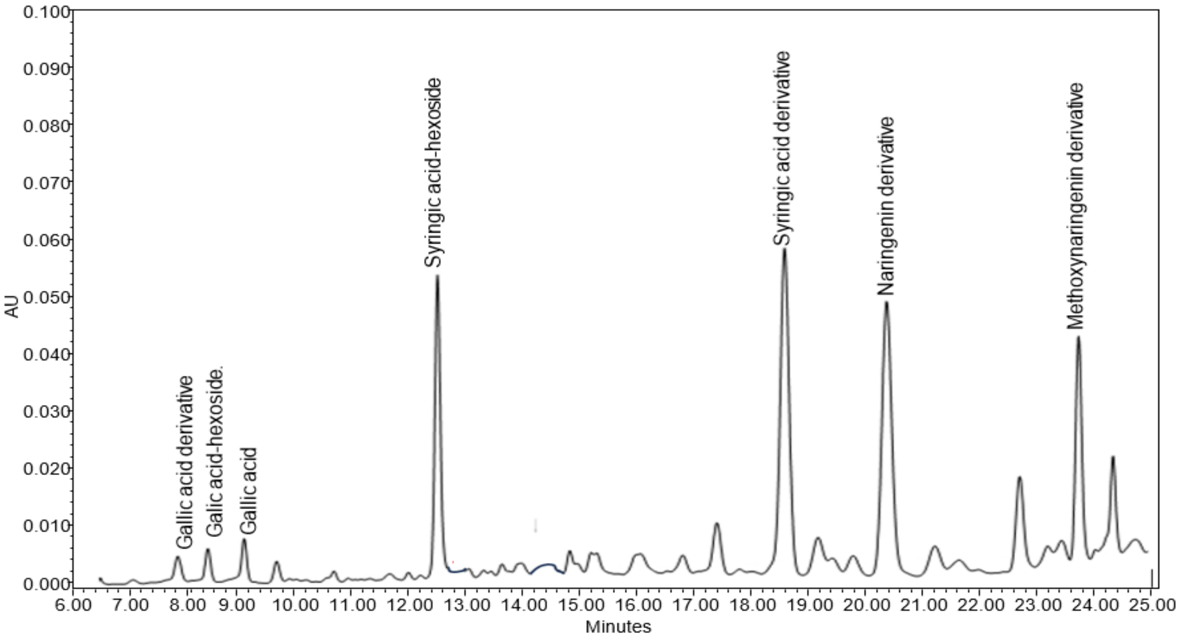

Figure S1 a: Compounds identified at 280 nm wavelength

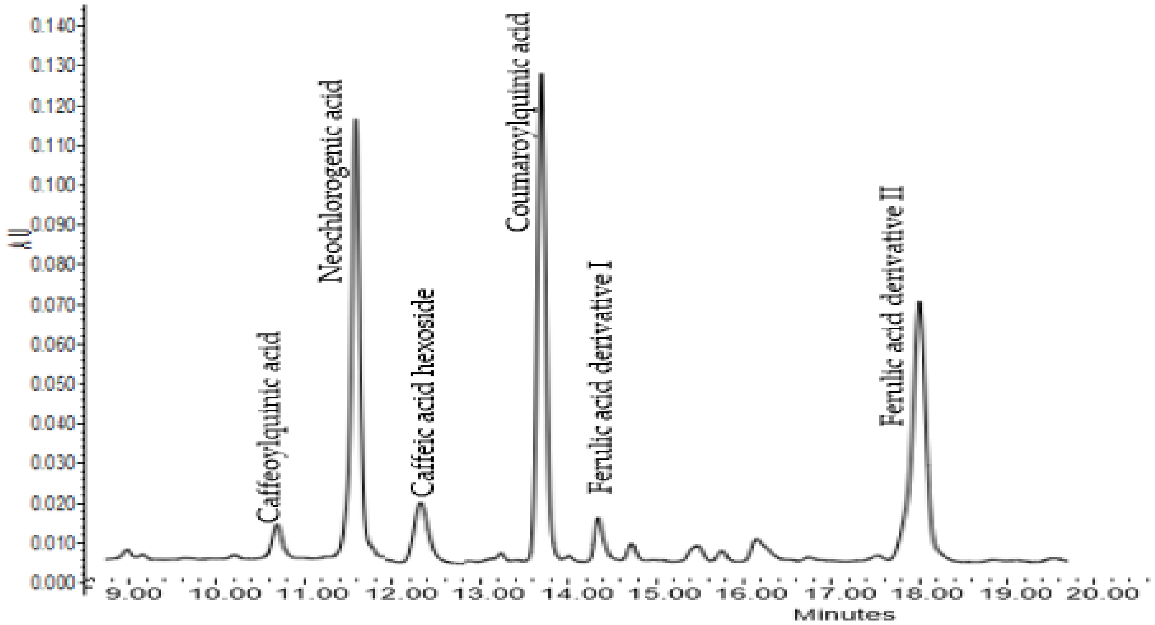

Figure S1 b: Compounds identified at 320 nm wavelength

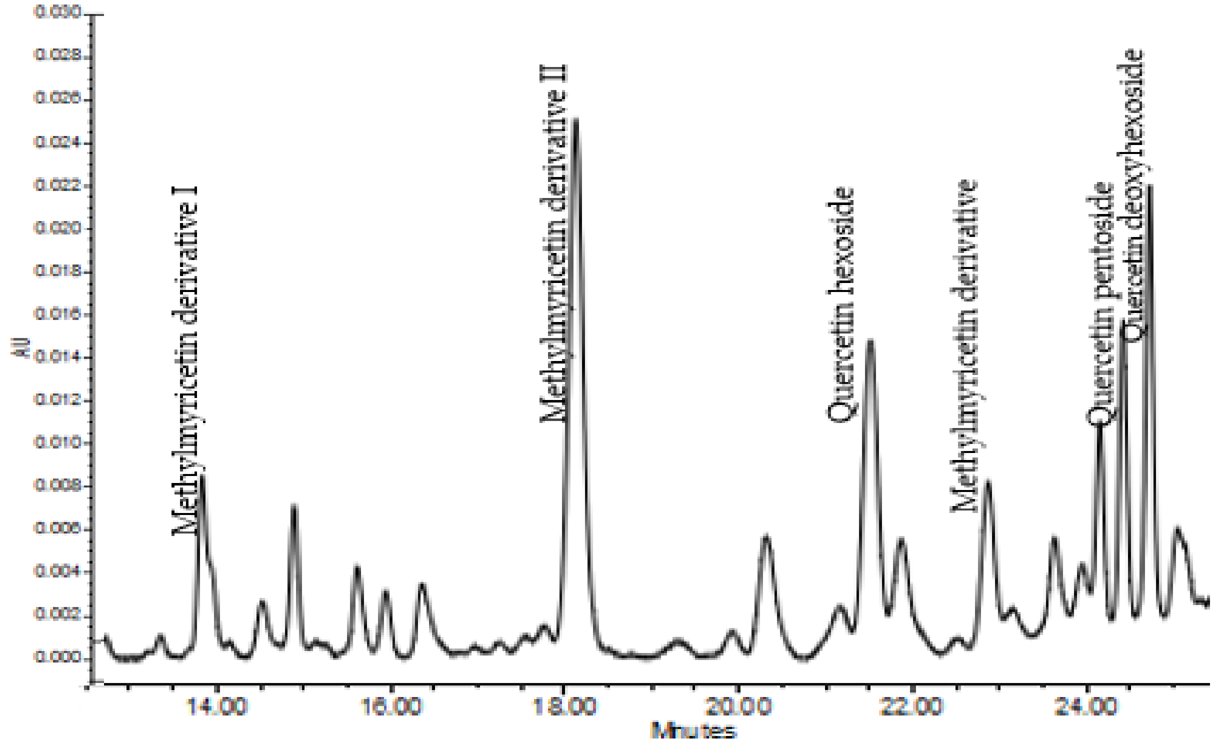

Figure S1 c: Compounds identified at 355 nm wavelength

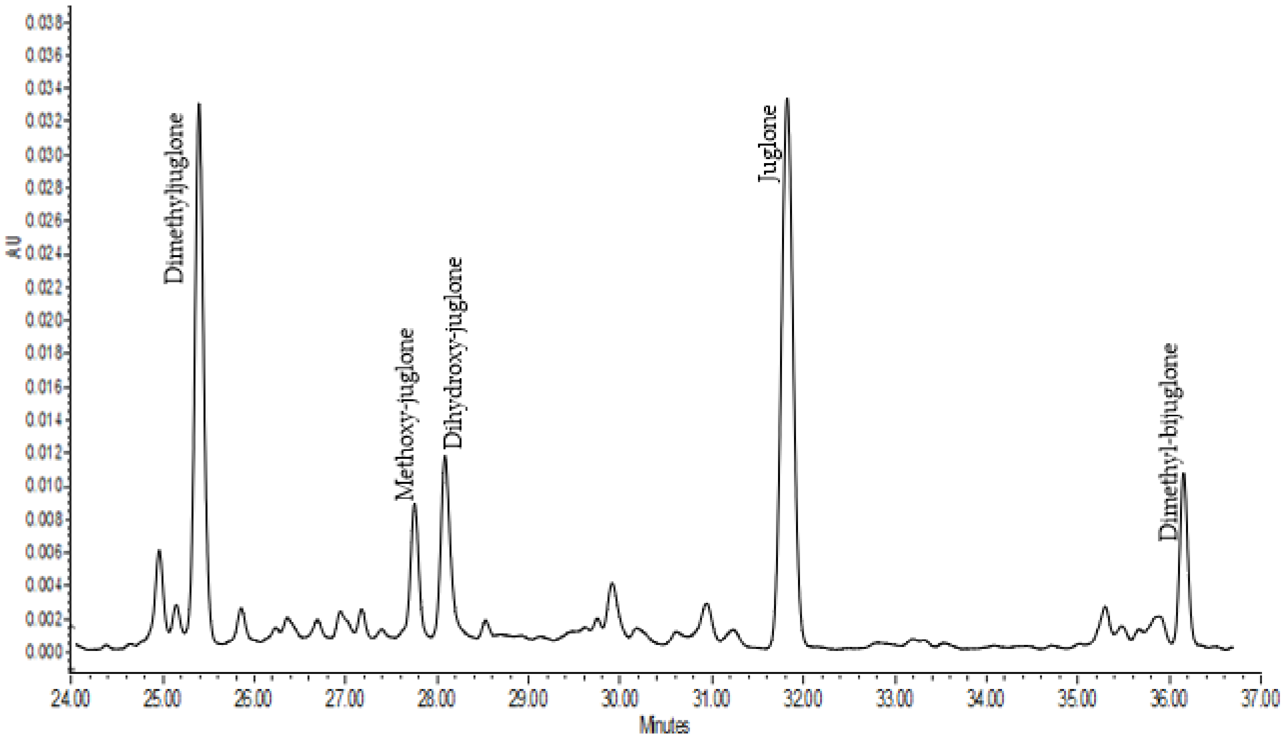

Figure S1 c: Compounds identified at 420 nm wavelength

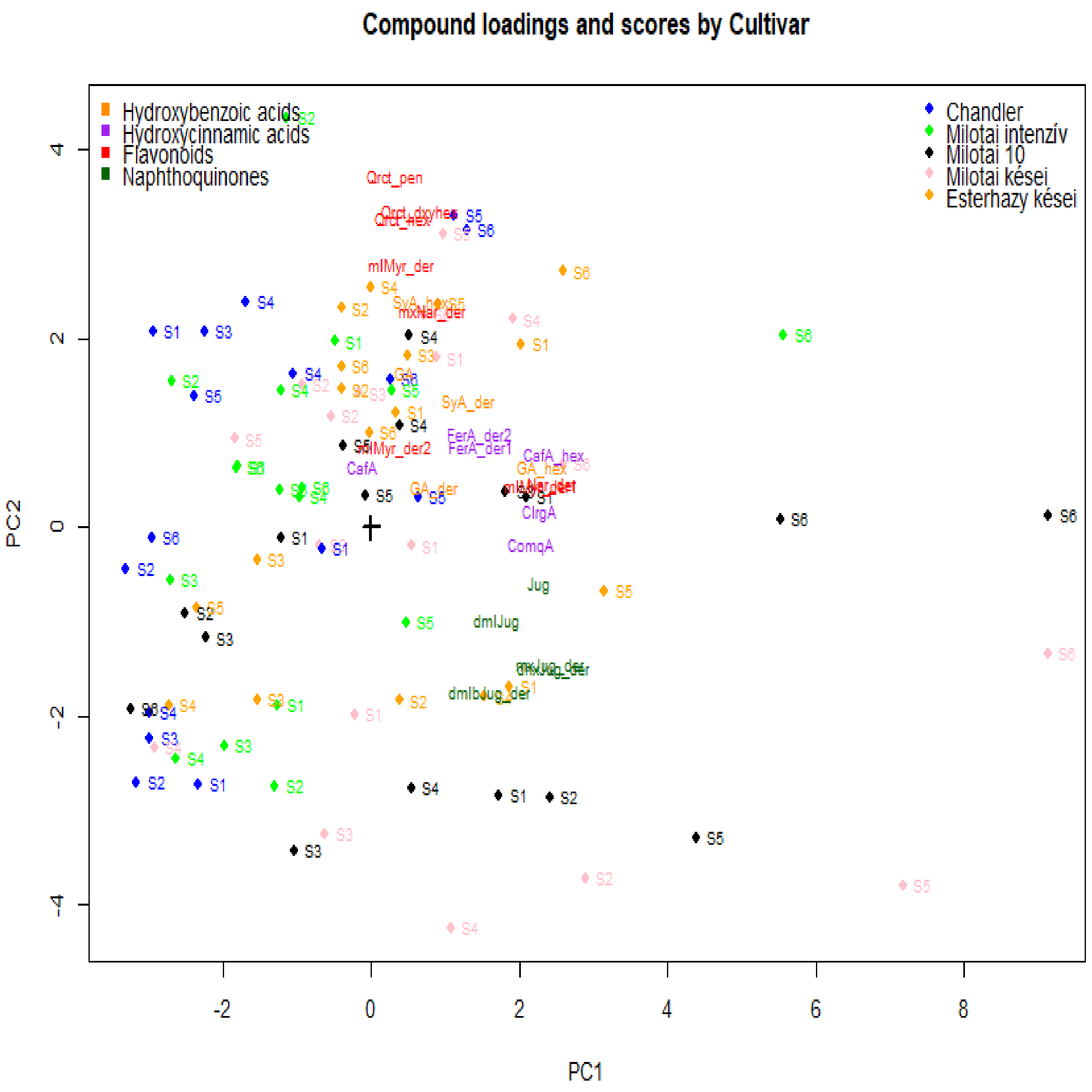

Figure S2: Biplot on phenolic loading and score clusters by Cultivar

Note: Points represent individual samples colored by cultivar; phenolic compounds are shown as loading labels scaled by a factor of six for graphical clarity. Therefore, interpretation focuses on the directions and relative positions of the compound.

Table S1: Factor Effect on log Phenolic acids and Flavonoids

Phenolic Compound	Term	estimate	std. error	*p*-value
Galic acid derivative	Intercept	2.877	0.265	0.000
	Esterhazy kései	1.618	0.318	0.000
	Milotai 10	0.640	0.321	0.050
	Milotai intenzív	0.370	0.321	0.254
	Milotai kései	0.172	0.318	0.590
	2023 (Y2)	-0.187	0.239	0.435
	2024 (Y3)	0.638	0.254	0.014
	Scaled (Cum_GDD)	-0.223	0.105	0.037
Galic acid-hexoside	Intercept	3.866	0.162	0.000
	Esterhazy kései	0.633	0.193	0.002
	Milotai 10	0.251	0.196	0.203
	Milotai intenzív	-0.026	0.196	0.894
	Milotai kései	0.440	0.193	0.026
	2023 (Y2)	-0.394	0.146	0.008
	2024 (Y3)	0.063	0.155	0.683
	Scaled (Cum_GDD)	0.138	0.064	0.034
Galic acid	Intercept	4.889	0.163	0.000
	Esterhazy kései	0.328	0.195	0.097
	Milotai 10	-0.450	0.197	0.025
	Milotai intenzív	-0.255	0.197	0.201
	Milotai kései	-0.409	0.195	0.039
	2023 (Y2)	-0.377	0.147	0.012
	2024 (Y3)	0.570	0.156	0.000
	Scaled (Cum_GDD)	0.107	0.064	0.102
Syringic acid-hexoside	Intercept	4.503	0.166	0.000
	Esterhazy kései	0.302	0.198	0.132
	Milotai 10	0.669	0.201	0.001
	Milotai intenzív	0.394	0.201	0.053
	Milotai kései	0.179	0.198	0.370
	2023 (Y2)	0.717	0.149	0.000
	2024 (Y3)	1.542	0.159	0.000
	Scaled (Cum_GDD)	0.095	0.066	0.152
Syringic acid derivative	Intercept	3.089	0.179	0.000
	Esterhazy kései	1.204	0.214	0.000
	Milotai 10	1.338	0.217	0.000
	Milotai intenzív	0.938	0.217	0.000
	Milotai kései	1.244	0.214	0.000
	2023 (Y2)	0.131	0.161	0.420
	2024 (Y3)	0.825	0.172	0.000
	Scaled (Cum_GDD)	0.010	0.071	0.886
Naringenin derivative	Intercept	5.257	0.126	0.000
	Esterhazy kései	0.772	0.150	0.000
	Milotai 10	0.700	0.152	0.000
	Milotai intenzív	0.237	0.152	0.124
	Milotai kései	0.770	0.150	0.000
	2023 (Y2)	-0.260	0.113	0.024

	2024 (Y3)	0.314	0.120	0.011
	Scaled (Cum_GDD)	-0.081	0.050	0.109
Methoxynaringenin derivative	Intercept	5.587	0.166	0.000
	Esterhazy kései	0.209	0.199	0.295
	Milotai 10	-0.576	0.201	0.005
	Milotai intenzív	-0.261	0.201	0.198
	Milotai kései	0.238	0.199	0.234
	2023 (Y2)	0.269	0.149	0.076
	2024 (Y3)	0.324	0.159	0.045
	Scaled (Cum_GDD)	0.186	0.066	0.006
Caffeoylquinic acid	Intercept	1.002	0.280	0.001
	Esterhazy kései	1.262	0.335	0.000
	Milotai 10	1.048	0.339	0.003
	Milotai intenzív	0.746	0.339	0.031
	Milotai kései	0.479	0.335	0.157
	2023 (Y2)	0.933	0.252	0.000
	2024 (Y3)	-0.097	0.268	0.719
	Scaled (Cum_GDD)	-0.251	0.111	0.027
Caffeic acid-hexoside	Intercept	3.852	0.118	0.000
	Esterhazy kései	0.322	0.141	0.025
	Milotai 10	0.371	0.143	0.011
	Milotai intenzív	0.189	0.143	0.191
	Milotai kései	0.268	0.141	0.061
	2023 (Y2)	-0.472	0.106	0.000
	2024 (Y3)	-0.001	0.113	0.996
	Scaled (Cum_GDD)	0.213	0.047	0.000
Neochlorogenic acid	Intercept	6.017	0.106	0.000
	Esterhazy kései	0.491	0.127	0.000
	Milotai 10	1.218	0.129	0.000
	Milotai intenzív	0.558	0.129	0.000
	Milotai kései	0.737	0.127	0.000
	2023 (Y2)	-0.254	0.096	0.009
	2024 (Y3)	0.144	0.102	0.160
	Scaled (Cum_GDD)	0.014	0.042	0.735
Coumaroylquinic acid	Intercept	6.113	0.227	0.000
	Esterhazy kései	0.395	0.272	0.151
	Milotai 10	0.753	0.275	0.008
	Milotai intenzív	0.358	0.275	0.197
	Milotai kései	1.049	0.272	0.000
	2023 (Y2)	-0.343	0.205	0.098
	2024 (Y3)	0.170	0.218	0.437
	Scaled (Cum_GDD)	0.012	0.090	0.894
Ferulic acid derivative I	Intercept	3.935	0.145	0.000
	Esterhazy kései	0.147	0.173	0.400
	Milotai 10	-0.159	0.176	0.369
	Milotai intenzív	0.001	0.176	0.995
	Milotai kései	0.286	0.173	0.103
	2023 (Y2)	-0.128	0.130	0.330

	2024 (Y3)	-0.111	0.139	0.426
	Scaled (Cum_GDD)	0.093	0.057	0.108
Ferulic acid derivative II	Intercept	6.292	0.345	0.000
	Esterhazy kései	1.675	0.413	0.000
	Milotai 10	0.777	0.418	0.067
	Milotai intenzív	0.021	0.418	0.960
	Milotai kései	0.498	0.413	0.231
	2023 (Y2)	-1.371	0.311	0.000
	2024 (Y3)	-1.075	0.331	0.002
	Scaled (Cum_GDD)	0.781	0.137	0.000
Methylmyricetin derivative	Intercept	3.492	0.272	0.000
	Esterhazy kései	-0.439	0.325	0.181
	Milotai 10	-0.283	0.329	0.392
	Milotai intenzív	0.163	0.329	0.622
	Milotai kései	-0.130	0.325	0.690
	2023 (Y2)	0.609	0.245	0.015
	2024 (Y3)	0.464	0.260	0.079
	Scaled (Cum_GDD)	0.187	0.108	0.086
Methylmyricetin derivative I	Intercept	2.985	0.320	0.000
	Esterhazy kései	0.473	0.383	0.221
	Milotai 10	0.211	0.388	0.588
	Milotai intenzív	0.397	0.388	0.310
	Milotai kései	0.784	0.383	0.044
	2023 (Y2)	-0.192	0.288	0.508
	2024 (Y3)	-0.864	0.307	0.006
	Scaled (Cum_GDD)	0.434	0.127	0.001
Methylmyricetin derivative II	Intercept	2.809	0.404	0.000
	Esterhazy kései	0.090	0.483	0.854
	Milotai 10	-0.545	0.489	0.269
	Milotai intenzív	-0.563	0.489	0.254
	Milotai kései	-0.174	0.483	0.719
	2023 (Y2)	-0.095	0.364	0.796
	2024 (Y3)	-0.879	0.387	0.026
	Scaled (Cum_GDD)	0.463	0.160	0.005
Quercetin hexoside	Intercept	5.864	0.170	0.000
	Esterhazy kései	-0.193	0.204	0.346
	Milotai 10	-0.559	0.206	0.008
	Milotai intenzív	0.030	0.206	0.883
	Milotai kései	-0.308	0.204	0.134
	2023 (Y2)	0.456	0.153	0.004
	2024 (Y3)	0.596	0.163	0.000
	Scaled (Cum_GDD)	0.256	0.067	0.000
Quercetin-pentoside	Intercept	4.805	0.132	0.000
	Esterhazy kései	-0.030	0.158	0.852
	Milotai 10	-0.197	0.160	0.221
	Milotai intenzív	0.025	0.160	0.877
	Milotai kései	-0.003	0.158	0.984
	2023 (Y2)	0.805	0.119	0.000
	2024 (Y3)	1.033	0.126	0.000

	Scaled (Cum_GDD)	0.072	0.052	0.170
Quercetin-deoxyhexoside	Intercept	4.348	0.160	0.000
	Esterhazy kései	-0.146	0.191	0.448
	Milotai 10	0.007	0.194	0.970
	Milotai intenzív	0.040	0.194	0.838
	Milotai kései	-0.088	0.191	0.645
	2023 (Y2)	0.854	0.144	0.000
	2024 (Y3)	0.757	0.153	0.000
	Scaled (Cum_GDD)	0.190	0.063	0.004

Notes: Estimates, standard errors and p-values from GLM with Gaussian error and identity link (GLM) fitted separately for each compound, with cultivar, year and standardized cumulative growing degree days (scaled Cum_GDD) as fixed effects; p-value < 0.05.
